# Clinical manifestations in children with parechovirus A and respiratory tract infection

**DOI:** 10.3389/fped.2026.1818273

**Published:** 2026-04-20

**Authors:** Lars Høsøien Skanke, Inger Heimdal, Hilde Lysvand, Nina Moe, Sidsel Krokstad, Andreas Christensen, Kari Risnes, Svein Arne Nordbø, Henrik Døllner

**Affiliations:** 1 Department of Clinical and Molecular Medicine, Norwegian University of Science and Technology (NTNU), Trondheim, Norway; 2 Department of Pediatrics, St. Olavs University Hospital, Trondheim, Norway; 3 Department of Medical Microbiology, St. Olavs University Hospital, Trondheim, Norway

**Keywords:** children, human rhinovirus, parechovirus A, respiratory syncytial virus, respiratory tract infection

## Abstract

**Background:**

The role of parechovirus A (PeV-A) in respiratory tract infections (RTI) in children remains unclear.

**Objectives and methods:**

We used clinical and virological data from two observational studies to study PeV-A in RTIs in children: a study of children admitted to hospital with RTI, and a study of children examined for RTI while attending day care centres. All had clinical examination and one nasopharyngeal aspirate analysed for PeV-A and 18 other viruses and bacteriae by culture and PCR-tests.

**Results:**

In the hospital study 4.6% (15/323) PeV-A positive RTIs were single virus detections. In 95.4% (308/323) other viruses were co-detected, including 28 with PeV-A and respiratory syncytial virus (RSV) and 77 with PeV-A and human rhinovirus (HRV). Multivariable logistic regression analysis showed strong association between single PeV-A and upper RTI (URTI) vs. lower RTI (LRTI) (age-adjusted OR 11.3, 95% CI 3.1−41.3). By retrospective evaluation of medical records, PeV-A was a likely cause of mainly pharyngitis and tonsillitis in 10/15 children with single PeV-A. In multivariable logistic regression modelling the presence of PeV-A had no impact on clinical manifestations and short-term outcomes in children with codetected RSV and HRV. In the day-care study PeV-A was detected in 30 children, among who 8/10 with single PeV-A had pharyngitis and tonsillitis.

**Conclusion:**

Single PeV-A detection was associated with pharyngitis and tonsillitis among children in day-care and hospital. Most hospitalized children with PeV-A had LRTI and viral codetections, but the presence of PeV-A did not impact disease severity in those with RSV and HRV.

## Introduction

The *Parechovirus A (PeV-A)* is one of 6 species in the *Parechovirus* genus, first described in 1999 (1). Since then, 19 genotypes of PeV-A have been proposed. PeV-A is increasingly recognized as a possible cause of infectious disease, primarily in children (2, 3), but also in adults (4). High prevalence of PeV-A neutralizing antibodies in serological samples suggests that most children are infected with PeV-A in early life (5–7). Whether these findings may be ascribed to symptomatic or asymptomatic infections is not clear (8). The most discussed clinical condition caused by PeV-A is sepsis-like disease in neonates, with or without signs of CNS infection (9–14). This sepsis-like disease is typically caused by PeV-A genotype 3 (15). PeV-A has also been associated with mild gastrointestinal symptoms, but asymptomatic fecal shedding of PeV has also been described (8, 16–18).

When it comes to respiratory tract infections (RTI) in children, we and others have reported an association between PeV-A detection in nasopharyngeal aspirate (NPA) and mild, self-limiting upper RTI (URTI), but few studies reported more detailed clinical findings (8, 19, 20). Moreover, the role of PeV-A in hospitalised children with severe RTI remains unclear. In a recent study, we found no significant difference in the prevalence of PeV-A between hospitalized Norwegian children with RTI and a comparison group of asymptomatic children undergoing elective surgery (8.8% vs. 10.1%) (21). Furthermore, codetection of other viruses were frequent in both the children with RTI (94%) and in elective controls (84%). Hence, we could not confirm a relation between PeV-A and RTI in hospitalized children (21).

In the present study, we aim to study the role of PeV-A in RTI using another approach with focus on the sick children only. For that purpose, we use two populations: 3552 Norwegian children admitted to hospital with RTI during a 10-year long period (21), and 161 children attending two day-care centres who were examined for symptoms and signs of RTI 2-4 times during a two-year long period (19). In both populations we identified children with PeV-A and described the clinical manifestations. Since viral co-detections were common in both populations, we have divided the analyses and study the impact of PeV-A in RTI in children with single PeV-A detection and in children with co-detected viruses.

## Materials and methods

### Hospital study

**Figure 1:**
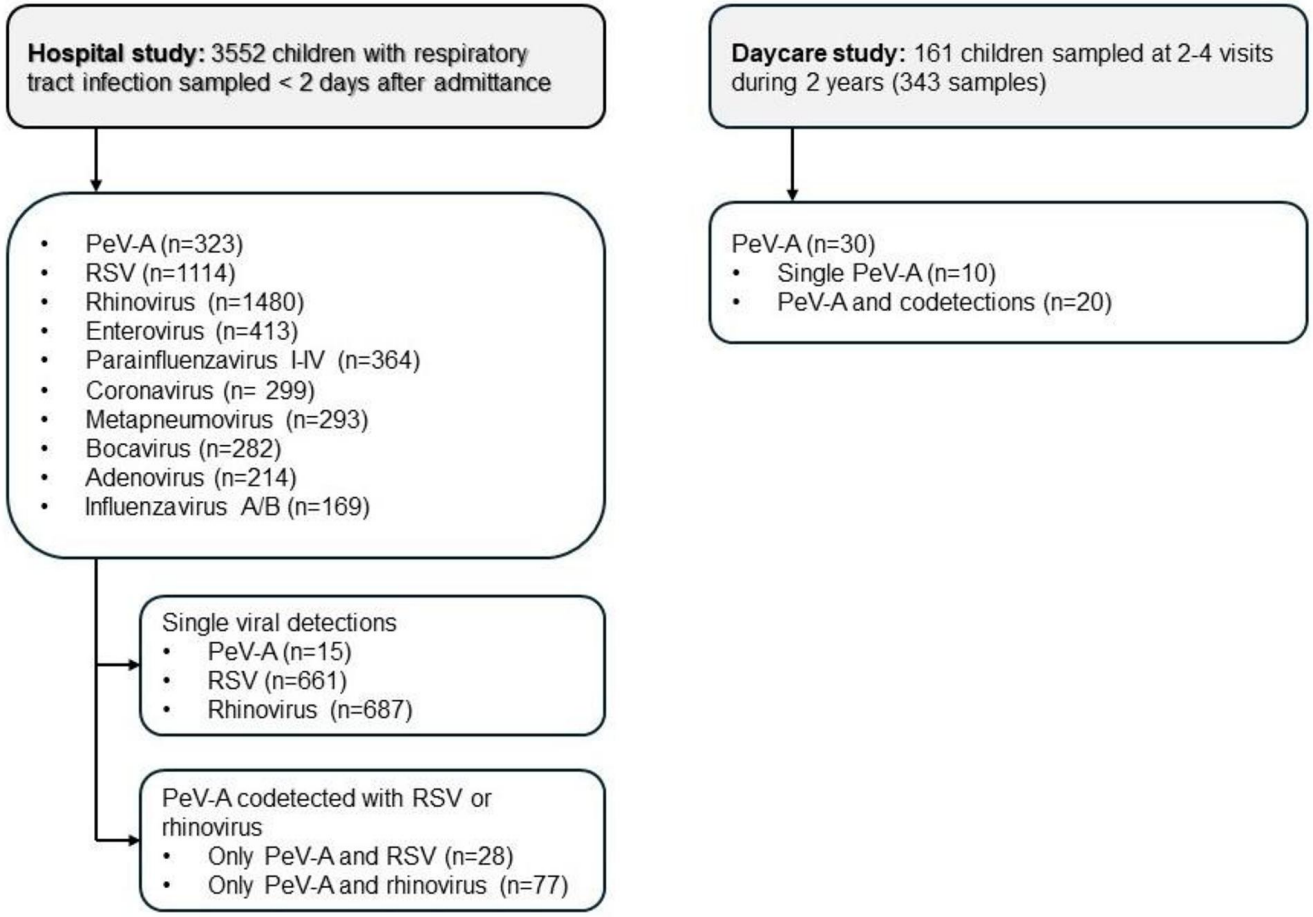
Flow-chart of the hospital study and the daycare study.

The hospital study has been described previously (21). In brief, children <16 years old referred for RTI and/or fever between November 2006 and July 2016, were prospectively enrolled at the Paediatric Department, St. Olav's Hospital, Trondheim University Hospital, Norway (*n* = 3552) (Figure 1). All children were sampled with one nasopharyngeal aspirate (NPA) for virus testing [PeV-A, human rhinovirus (HRV), respiratory syncytial virus (RSV) and others]. Exclusion criteria were: 1) children undergoing chemotherapy or immunosuppressive treatment and 2) admissions with absence of a valid test results for PeV-A, HRV or RSV. All children were treated at the discretion of the medical doctors who were also aware of virus test results.

Children were retrospectively divided into 2 groups: URTI and lower RTI (LRTI). URTI included children with rhinitis, rhinosinusitis, stomatitis, pharyngitis, laryngitis, otitis media or tonsillitis without signs of LRTI. Lower RTI was diagnosed in the presence of dyspnea, lower airway obstruction, or chest x-ray infiltrates, with or without signs of URTI. We defined chronic disease as the presence of either epilepsy, cerebral palsy, asthma, immunodeficiency, congenital heart disease or congenital anomalies.

Two independent paediatric authors (LHS and HD) evaluated the medical record of each child with PeV-A as sole viral finding, and classified PeV-A as a likely, a possible or an unlikely cause of the RTI, based on clinical experience. Any discrepancy between the authors was solved by consensus. We then studied if PeV-A was associated with clinical manifestations and disease severity in children with RTI who had codetection with RSV or HRV only, the two most common respiratory viruses. We compared children with RSV+PeV-A to children with single RSV, and children with HRV+PeV-A to children with single HRV, respectively. In these analyses, we used a severity score previously used in other studies (22), with a maximum of 10 points. One to six points were given for respiratory support: 1 point for oxygen, 2 points for High Flow Nasal Cannula (HFNC), 3 points for Continuous Positive Airway Pressure (CPAP) or Bi-level Positive Airway Pressure (BiPAP), 4 points for Non-Invasive Ventilation (NIV) other than CPAP and BiPAP and 6 points for invasive respirator with endotracheal intubation. Two points were given for fluid replacement treatment by intravenous route or nasogastric tube, and 2 points were given for hospital stay 5 days or longer, corresponding to the 75-percentile for hospital stay.

### Study of children attending day care

We used data from a previously published study from two Norwegian day care centres for children, performed to assess how often respiratory viruses may appear in children during everyday life (19). A day care centre is a facility providing care for pre-school children during every-day life. Individual children were included from 2 to 4 times during four visits to the day care centres in 2013-14. The caretakers provided information about current health status, a nasopharyngeal swap was collected and paediatricians examined all children using these criteria in the classification: 1) Mild URTI with discrete signs of rhinitis, pharyngitis, otitis media and/or secretory otitis; 2) URTI with significant signs of rhinopharyngitis, tonsillitis and/or purulent media otitis; 3) No signs of RTI. In the present work, we included all children with an URTI and detection of PeV-A without (*n* = 10) and with (*n* = 20) codetections of other viruses.

### Samples and laboratory methods

The NPAs were analysed for PeV-A and 15 other respiratory viruses, *Bordetella pertussis, Chlamydia pneumoniae* and *Mycoplasma pneumoniae* by use of in-house RT-PCR testing (19, 21, 23). NPAs from children in the hospital study were also aerobe cultured, and PeV-A positive samples were genotyped, as previously described (21). Blood samples were collected in the hospital study and analysed for white blood cell counts and C-reactive protein.

### Statistical analysis

We used standard descriptive measures and hypothesis testing with *χ*^2^ test or Fishers exact test, Mann–Whitney U test and Student's t test, as appropriate. We used multivariable logistic regression analysis to study the associations between URTI (as opposed to LRTI) and single PeV-A detection (as opposed to PeV-A + every viral codetection), and between single PeV-A and PeV-A Ct-values, respectively, adjusting for age (months) in both analyses. The same method was used to assess the relation between severe disease and the presence of PeV-A in children with RSV and HRV, respectively. A total severity score of ≥3 was used as an indicator of severe disease, corresponding to the 75-percentile of the severity score in the entire group of admitted children. We adjusted the analysis for age (months). Children with both RSV and HRV or other codetected viruses were excluded from the analysis. We reported odds ratios (OR) or adjusted odds ratios (aOR) and 95% confidence intervals (CI) as strength of the associations. For all tests, a *p*-value <0.05 was considered statistically significant. All analyses were performed using IBM SPSS Statistics 27.

### Ethical approval and consent to participate

The study performance adhered to the Declaration of Helsinki. The studies were approved by the Regional Committees for Medical and Health Research Ethics, Central Norway in 2006 (No: 06/2289), 2008 (No: 08/2142), 2011 (No: 11/2246) and 2012 (No: 12/1042). In the hospital study, children admitted with respiratory infections were enrolled after written and oral informed consent from their parents or legal guardians. Some of the children were discharged before enrolment. They were instead informed about the study by a postal letter after hospital discharge, and their parents or legal guardians could reserve against enrolment by contacting the researchers in 2 weeks. In the day-care study caregivers or legal guardians to all the children received written and oral information and written consent to participate was collected.

## Results

### Hospital study

Virus findings in NPA samples are shown in Figure 1. PeV-A was detected in 323 (9.1%) of 3552 children with RTI, among whom only 15 (4.6%) had single PeV-A and 308 (95.4%) had one or more codetected viruses. RSV and HRV were detected in 1114/3552 (31%) and 1480/3552 (42%), respectively (Figure 1).

### Hospital study: clinical manifestations in children with single PeV-A detection

Median age of the 15 children with single PeV-A was 13.4 months (IQR 7.9 months). Nasal congestion and secretion, cough and pharyngeal inflammation and soreness were the dominating signs and symptoms (Table 1). Eleven children (73%) were diagnosed with an URTI including rhinitis (*n* = 1), pharyngitis (*n* = 4), tonsillitis (*n* = 4), laryngitis (*n* = 1) and stomatitis (*n* = 1). Three children (20%) had a LRTI (one child with pneumonia and two with bronchiolitis), and one child had fever without a clear focus. Six of the 15 children also had otitis media, but this was not regarded as the main infection in any of the cases. Eight children had PeV-A1, 2 had PeV-A3, 1 had PeV-A6 and sequencing failed in 4 (Table 1). A logistic regression analysis showed a strong association between URTI and single PeV-A detection (age adjusted OR 11.3, 95% CI 3.1-41.3), but single PeV-A was not related to higher PeV-A genomic loads (age adjusted OR 1.0, 95% CI 0.9-1.1). Aerobe cultures in NPA were dominated by *Streptococcus pneumoniae, Haemophilus influenza* and *Moraxella catarrhalis* (Table 1). After evaluation by 2 independent pediatricians (LHS and HD), we found that in 10 of 15 (67%) children, PeV-A was a likely contributing factor to the infection, and in 4 of 15 (27%), PeV-A had a possible contribution (TTable 1).

**Table 1:** Findings in 15 hospitalized children with respiratory tract infections and detection of single parechovirus A (PeV-A) in nasopharyngeal aspirate.

Child	Age, months	Nasal congestion - secretion	Signs of otitis	Pharyngal inflam- mation	Tonsillar inflam-mation and pus	Conjunctival inflammation	Cough	Wheeze	Ronchi	Temperature ( °C)	CRP (mg/L)	WBC (x10^9^/L)	Main diagnosis	Bacterial growth^a^	PeV-A genotype	PeV-A as an agent contributing to the infection^b^
1	0.6	+	-	+	-	-	-	-	-	39.0	<5	7.6	Rhinitis	*Normal flora*	PeV-A3	*Likely*
2	6.5	-	-	+	-	-	+	-	-	37.0	NA	NA	Pharyngitis	*Not cultured*	PeV-A1	*Likely*
3	10.5	+	-	-	-	+	+	-	-	38.2	129	15	Laryngitis	*No growth*	PeV-A3	*Likely*
4	12.2	-	-	+	+	-	-	-	-	39.4	<5	15.2	Tonsillitis	*SA, MC*	PeV-A1	*Likely*
5	13.4	+	-	+	-	-	+	-	-	38.2	142	13.8	Pharyngitis	*SP, HI*	PeV-A6	*Possible*
6	15.0	-	+	+	-	-	-	-	-	40.4	<5	3.2	Pharyngitis	*SP, MC*	Failed	*Possible*
7	15.2	+	+	+	-	-	-	-	-	36.7	31	8.8	Pharyngitis	*SP*	Failed	*Likely*
8	15.4	-	-	+	-	-	-	-	-	38.3	10	7.7	Stomatitis	*NHS*	PeV-A1	*Likely*
9	15.8	+	-	+	+	+	+	-	-	39.4	54	11.5	Tonsillitis,			
cellulitis	*No growth*	PeV-A1	*Likely*													
10	17.3	-	+	+	+	-	-	-	-	40.4	203	18.6	Tonsillitis	*No growth*	PeV-A1	*Possible*
11	30.2	+	-	+	+	-	-	-	-	38.4	311	30	Tonsilitis	*MC*	Failed	*Possible*
12	7.5	-	+	-	-	-	-	-	+	36.9	37	13.9	Pneumonia	*SA*	PeV-A1	*Unlikely*
13	7.9	+	+	+	-	-	+	-	+	40.0	28	9.3	Bronchiolitis	*SP, HI, MC*	PeV-A1	*Likely*
14	9.8	+	-	-	-	-	+	+	+	37.1	11	14.4	Bronchiolitis	*SA, HI*	Failed	*Likely*
15	5.2	-	-	-	-	-	-	-	-	37.7	<5	12.1	FUO	*EC*	PeV-A1	*Likely*

a Bacterial growth in upper airway secrete: *Staphylococcus aureus* SA, *Haemophilus influenzae* HI, *Moraxella catarrhalis* MC, *Streptococcus pneumonia* SP, *non-hemolytic streptococcus* NHS, *E.coli* EC.

b PeV-A as a likely, possible, or unlikely contributor to disease, evaluated by two independent authors. FUO, fever of unknown origin.

### Hospital study: does codetected PeV-A impact clinical manifestations and disease severity in children with either RSV or HRV?

Overall, there were few differences in clinical manifestations, laboratory measurements, diagnoses and treatments between the children with RSV+PeV-A (*n* = 28) and children with single RSV (*n* = 661), but children with RSV + PeV-A were older than those with single RSV (Table 2). Less children with RSV + PeV-A had a severe disease defined as a severity score ≥3 compared to children with single RSV (28.6% vs. 41.8%), but this difference was not statistically significant (Table 2) and an age-adjusted multivariable logistic regression analysis confirmed that PeV-A co-detection was not associated with severe disease (aOR 0.55, 95% CI: 0.24-1.28). Comparing children with HRV + PeV-A (*n* = 77) to children with single HRV (*n* = 687), there were neither any differences in clinical manifestations, laboratory measurements, diagnoses or treatments associated with PeV-A codetection (Table 2). Moreover, no differences in disease severity were found whether for single factors or the severity score (aOR 1.30, 95% CI 0.70-2.41)(Table 2).

**Table 2:** Comparison of hospitalized children with respiratory tract infections and RSV, HRV and PeV-A detection in nasopharyngeal aspirate.

Characteristics	Single RSV (*n* = 661)	RSV + PeV-A (*n* = 28)	*p*-value	Single rhinovirus (*n* = 687)	Rhinovirus + PeV-A (*n* = 77)	*p*-value
Age, median (IQR) (months)	5.6	14.5	<.001	15.3	15.8	.07
Premature birth	81 (12.3%)	2 (7.4%)	.76	84 (13.4%)	14 (18.4%)	.22
Chronic disease^a^	83 (12.6%)	4 (14.3%)	.77	152 (22.1%)	16 (20.8%)	.61
Sex, male	360 (54.5%)	20 (71.4%)	.08	450 (65.5%)	48 (62.3%)	.63
Peak temperature (°C)	38.1	38.7	.13	37.8	37.8	1.0
Median C-Reactive Protein (mg/L)	14	37	.07	12	18	.42
Median WBC (x10^9^/L)	10.8	10.1	.72	13.1	15.4	.47
X-ray infiltrate	304/369 (82.4%)	17/17 (100%)	.14	184/333 (55.3%)	25/41 (61%)	.49
Retractions	478 (72.3%)	16 (57.1%)	.08	446 (64.9%)	42 (54.5%)	.07
Wheezing	402 (60.8%)	20 (71.4%)	.53	427 (62.2%)	48 (62.3%)	.98
Signs of otitis media	135 (20.4%)	11 (39.4%)	.017	116 (16.9%)	17 (22.1%)	.25
Pharyngeal inflammation	219 (33.1%)	12 (42.9%)	.29	225 (32.8%)	32 (41.6%)	.12
Nasal congestion - secretion	225 (34.0%)	10 (35.7%)	.86	249 (36.2%)	32 (41.6%)	.36
Upper RTI^b^	27 (4.1%)	0 (0%)	.28	184 (26.8%)	19 (24.7%)	.69
Lower RTI^c^	634 (95.9%)	28 (100%)	.28	503 (73.2%)	58 (75.3%)	.69
- Pneumonia	105 (16.6%)	4 (14.3%)		52 (7.6%)	7 (9.1%)	
- Bronchiolitis	485 (76.5%)	22 (78.6%)	.59	233 (33.9%)	28 (36.4%)	.41
- Unspecified lower RTI	44 (6.9%)	2 (7.1%)		218 (43.3%)	23 (29.9%)	
Antibiotics	151 (23.2%)	8 (28.6%)	.17	112 (16.9%)	17 (23%)	.19
Inhalations	622 (94.2%)	26 (92.9%)	.61	532 (77.8%)	57 (76%)	.13
Corticosteroids	138 (21.1%)	9 (32.1%)	.06	306 (45.2%)	36 (48.6%)	.57
LOS (median)(days)	4	3	.15	2	2	.19
LOS ≥5 days	244 (36.9%)	7 (25%)	.20	111 (16.2%)	11 (14.3%)	.67
Fluid support	247 (37%)	10 (35.7%)	.58	113 (16.4%)	13 (16.9%)	.41
Oxygen nasal	300 (45.4%)	9 (32.1%)		218 (31.7%)	25 (32.5%)	
High humidified flow nasal cannula	32 (4.8%)	1 (3.6%)		18 (2.6%)	1 (1.3%)	
Continous positive airway pressure	42 (6.4%)	2 (7.1%)	.40	20 (2.9%)	3 (3.9%)	.73
Non-invasive ventilation (with overpressure)	10 (1.5%)	1 (3.6%)		4 (0.4%)	0 (0%)	
Invasive ventilation	15 (2.3%)	1 (3.6%)		3 (0.4%)	0 (0%)	
Severe disease^d^	276 (41.8%)	8 (28.6%)	.17	109 (15.9%)	14 (18.2%)	.41

LOS, length of stay; RTI, respiratory tract infection; RSV, respiratory syncytial virus; HRV, human rhinovirus; PeV-A, parechovirus-A.

a Chronic disease includes asthma, cerebral palsy, epilepsy, immunodeficiency and heart disease.

b URTI: Clinical manifestations of rhinitis, pharyngitis, otitis media or tonsillitis.

c LRTI: Clinical manifestations of bronchiolitis, pneumonia or unspecified lower RTI.

d Severe disease: A score of ≥3 in a severity score based on need of oxygen, respiratory support, need for fluid replacement and length of stay.

### Children with PeV-A in day-care

PeV-A positive children (*n* = 30) were younger compared to the entire day-care population who were sampled (PeV-A positive: median age 22.5 months, IQR 13 months, PeV-A negative: median age 42 months, IQR 28 months, *P* < .001.) and all but 4 (87%) with PeV-A were younger than 36 months old. PeV-A was present as a single detection in 10 and codetected with other viruses in 20 children (Table 3). Eight children (80%) with single PeV-A had URTI, among who 3 (30%) were evaluated by clinical examination to be mild and 5 (50%) were evaluated to have a URTI with more significant signs of rhinitis and pharyngitis (Table 3).

**Table 3:** Clinical manifestations and viral codetections in children attending day-care with detection of parechovirus-A (PeV-A) in nasopharyngeal aspirate.

Child	Nasal congestion or secretion	Mild signs of otitis media	Purulent otitis media	Pharyngeal inflammtion	Conjunctival inflmmation	Viral codetection^a^	Clinical classification^b^
1	+	NE	NE	NE	-	-	Mild URTI
2	+	-	-	-	-	-	Mild URTI
3	+	NE	NE	NE	-	-	Mild URTI
4	+	-	-	+	-	-	URTI
5	+	-	-	+	+	-	URTI
6	+	-	-	+	-	-	URTI
7	+	+	-	+	-	-	URTI
8	+	+	-	+	-	-	URTI
9	-	-	-	-	-	-	No RTI
10	NE	NE	NE	NE	NE	-	NE
11	+	+	-	-	-	EV	Mild URTI
12	-	+	-	+	-	EV	Mild URTI
13	+	+	-	-	-	HRV	Mild URTI
14	-	+	-	-	-	EV	Mild URTI
15	-	-	-	-	-	HRV	Mild URTI
16	+	+	-	NE	-	EV, HRV	URTI
17	+	-	+	-	-	HBoV, HRV	URTI
18	+	+	-	-	-	HRV	URTI
19	+	+	-	-	-	HCoV, HRV	URTI
20	+	+	-	+	-	HBoV, EV, HRV	URTI
21	+	+	-	-	-	EV, HRV	URTI
22	+	+	-	-	+	HRV	URTI
23	+	+	-	+	-	EV	URTI
24	+	+	-	+	-	PIV4	URTI
25	+	NE	NE	+	-	EV	URTI
26	-	-	-	-	-	HRV	No RTI
27	-	NE	NE	NE	-	HRV	No RTI
28	-	-	-	-	-	HBoV, PIV4, HRV	No RTI
29	-	NE	NE	NE	-	EV	No RTI
30	-	NE	NE	-	-	HRV	No RTI

a Viral codetection: HBoV, human bocavirus; HCoV, human coronavirus; EV, enterovirus; PIV4, parainfluenza virus 4; HRV, human rhinovirus.

b Upper respiratory tract infection (URTI) was classified by paediatricians after clinical examination as 1; Mild URTI with *discrete signs* of rhinitis, pharyngitis, otitis media and/or secretory otitis. 2; URTI with *significant signs* of rhino-pharyngitis, tonsillitis and/or purulent media otitis. 3; No signs of RTI. NE: Refused examination.

Among the 20 children with PeV-A and viral codetections (Table 3), 5 (25%) were evaluated to have a mild URTI with discrete signs of rhinitis (*n* = 2), pharyngitis (*n* = 1), and otitis media (*n* = 4), and 10 (50%) had a URTI with significant rhinitis (*n* = 10), pharyngitis (*n* = 4) and purulent otitis media (*n* = 1). HRV (*n* = 13) and enterovirus (*n* = 9) were most often codetected. There was no difference in PeV-A genomic loads in children with single PeV-A and PeV-A with codetections (mean Ct-value 35 vs. 34, *p* = .45).

## Discussion

We found that PeV-A is associated with common upper respiratory tract infections in mostly less than 3 years old children, ranging from children in normal vigour with discrete signs and symptoms attending day-care to infants with more significant manifestations occasionally in need of hospitalization. However, it is striking that most children with PeV-A were coinfected with RSV, HRV and other respiratory viruses, and we could not show that PeV-A had any impact on clinical manifestations and disease severity in children with RSV and HRV at the hospital.

Data from the day care study showed 8 well-appearing children with clinical signs and symptoms of non-febrile common cold and single PeV-A detection, as examples of what might be a common presentation of PeV-A infection among children in the community. In the hospital population only 15 out of 323 (4.6%) PeV-A positive samples collected during a ten-year long period were single PeV-A detections, among who PeV-A was a likely contributor in 8 children with rhinitis, pharyngitis, tonsillitis, laryngitis and stomatitis and 2 children with bronchiolitis. Previously, we compared the entire hospital population of 323 PeV-A positives with RTI with a group of asymptomatic hospital controls where 10.1% had PeV-A, and found no association between PeV-A in nasopharyngeal aspirate samples and RTI (21). However, this approach comparing cases and controls may be criticized in research using gene technology tools because nucleic acids may be detected long after active infection giving rise to high detection rates in asymptomatic children. The data from the present study are based entirely on the RTI group, and with this approach, we suggest that PeV-A may be associated with various URTIs in a few hospitalized children with single-PeV-A. Taken together our findings suggest that PeV-A in children may be associated with a broad clinical spectrum of URTIs and a few children in need of hospitalization. However, in the large group of children admitted to hospital with LRTI, we found that PeV-A has a limited significance. A study from Hongkong using a PCR-panel with fewer virus types than we did has previously described children with single PeV-A and clear signs of URTI and a few children with bronchiolitis (20). PeV-A has been linked to outbreaks of RTI in infants at neonatal units (24) and to children with otitis media (25). However, studies from Scotland (26) and Italy (27) detected low PeV-A prevalence and did not find any clear association with RTI, and the association with otitis media has been questioned by a study from Finland (28). Most of previous studies have been hospital-based (3), but recently in a longitudinal cohort study from Australia following 130 newborns in the community and where parents reported respiratory symptoms (8), the authors found an association between PeV-A detection in stools samples and/or nasal swabs and upper respiratory symptoms, although most children had viral codetections of rhinovirus and other respiratory viruses (8). Indeed, frequent viral co-detections often challenge the interpretation of causal relations between a specific virus and clinical manifestations. To the best of our knowledge, our study is the first to systematically evaluate PeV-A as a codetected virus in children with the 2 most common respiratory viruses in children, RSV and rhinovirus, and it is an important finding that we could not reveal any impact of PeV-A over RSV and rhinovirus on clinical manifestations and short-term outcomes. However, with our methods we can never exclude the possibility that in some children with LRTI, PeV-A occasionally may contribute to symptoms e.g., from the upper airways. PeV-A detection in nasopharyngeal aspirates may also represent asymptomatic infection and PCR-tests detecting nucleic acids may always indicate long-term viral shedding after former RTI (8, 29). Among the hospitalized children with RTI and single PeV-A detection in our study, some had very high CRP-levels suggesting combined viral-bacterial infection, but it is not possible to clarify this important aspect using our data based on a cross-sectional study design.

We have previously shown that PeV-A and subtypes PeV-A1 and PeV-A6 have a seasonal circulation (21) and are frequently detected in airway samples from infants and children hospitalized with RTIs, but compared to healthy controls the virus is not associated with RTIs (19). In the present study we show that PeV-A may be related to URTIs in children, but due to the use of sensitive PCR tests PeV-A may often be co-detected in other viral infections such as RSV and HRV. PeV-A3 is associated with sepsis-like disease and encephalitis in infants (10–13) and this genotype causes severe disease as compared to PeV-A subtypes associated with URTIs. A future prevention strategy should search for better understanding of the pathogenesis in PeV-A and particularly PeV-A3 infections, and the development of an effective and affordable vaccine should be studied.

It is a strength of the present studies that we examined all children clinically, including children in the day-care centre. The prospective inclusion over a 10-year long period in the hospital study with systematic assessment of clinical information and the use of the same in-house PCR tests for a broad panel respiratory viruses are strengths to support the findings, minimising bias from seasonal variations and spectrum bias. There are several limitations. The observational study design is prone to selection bias; it is neither suitable to study the impact of viral and bacterial codetections and precludes assumptions about causality. The day-care study had a limited size, and both studies were performed at one centre, but the hospital study at St. Olavs University hospital cover a broad spectrum of children because the hospital receives all children in need of paediatric service from the entire Sør-Trøndelag county in Mid-Norway. The low number of children with single PeV-A detection is a limitation, on the other hand, the study documents that viral codetections are very common and should be accounted for in the evaluation of clinical manifestations. In the absence of a valid scoring tool, in this and other studies we used a non-validated clinical severity score in the evaluation of codetected viruses (29). However, it is composed of central clinical variables reflecting clinical routines at our hospital, and analyses of each clinical variable came to the same conclusions. It is also a limitation that genotyping of PeV-A and bacterial cultures were not performed in the day care studyt Finally, in the classification of clinical findings no objective guidelines were followed, only clinical judgement by experienced paediatric consultants.

## Conclusion

We conclude that single PeV-A is a likely contributor to mild and more severe cases of pharyngitis, tonsillitis and other URTIs in children, and is rarely involved in LRTIs. Most children with PeV-A have viral codetections, and it is not possible to demonstrate any impact of PeV-A in hospitalized children with RTI associated with RSV and HRV.

## Data Availability

The data are available from Central Norway Regional Health Authority's IT department (Hemit), but restrictions apply to the availability of these data, which were used under license for our research group, and so are not publicly available. Data are however available from the authors upon reasonable request and with permission of Regional Committees for Medical and Health Research Ethics, Central Norway.. Requests to access the datasets should be directed to Henrik Døllner, henrik.dollner@ntnu.no.
